# Map-based cloning and characterization of *BPH29*, a B3 domain-containing recessive gene conferring brown planthopper resistance in rice

**DOI:** 10.1093/jxb/erv318

**Published:** 2015-07-01

**Authors:** Ying Wang, Liming Cao, Yuexiong Zhang, Changxiang Cao, Fang Liu, Fengkuan Huang, Yongfu Qiu, Rongbai Li, Xiaojin Lou

**Affiliations:** ^1^State Key Laboratory of Genetic Engineering, Institute of Genetics, School of Life Sciences, Fudan University, Shanghai 200438, China; ^2^Crop Breeding and Cultivation Research Institute, Shanghai Academy of Agricultural Sciences, Shanghai 201403, China; ^3^State Key Laboratory for Conservation and Utilization of Subtropical Agro-bioresources and College of Agriculture, Guangxi University, Nanning 530004, China; ^4^Plant Protection Research Institute, Guangxi Academy of Agricultural Sciences, Nanning 530007, China

**Keywords:** B3 domain protein, *BPH29*, brown planthopper resistance, map-based cloning, *Oryza sativa* L., plant–insect defence, recessive gene.

## Abstract

Rice (*Oryza sativa* L.) production, essential for global food security, is threatened by the brown planthopper (BPH). The breeding of host-resistant crops is an economical and environmentally friendly strategy for pest control, but few resistance gene resources have thus far been cloned. An *indica* rice introgression line RBPH54, derived from wild rice *Oryza rufipogon*, has been identified with sustainable resistance to BPH, which is governed by recessive alleles at two loci. In this study, a map-based cloning approach was used to fine-map one resistance gene locus to a 24kb region on the short arm of chromosome 6. Through genetic analysis and transgenic experiments, *BPH29*, a resistance gene containing a B3 DNA-binding domain, was cloned. The tissue specificity of *BPH29* is restricted to vascular tissue, the location of BPH attack. In response to BPH infestation, RBPH54 activates the salicylic acid signalling pathway and suppresses the jasmonic acid/ethylene-dependent pathway, similar to plant defence responses to biotrophic pathogens. The cloning and characterization of *BPH29* provides insights into molecular mechanisms of plant–insect interactions and should facilitate the breeding of rice host-resistant varieties.

## Highlight

A brown planthopper resistance recessive gene, *BPH29*, was cloned which contained a B3 DNA-binding domain and conferred resistance by a mechanism that was similar to plant defence against pathogens.

## Introduction

Rice (*Oryza sativa* L.), an important staple food for >3 billion people worldwide (Khush, 1997), is currently beset by multiple threats. Plant domestication has presumably narrowed crop genetic diversity and decreased resistance to abiotic and biotic stresses, leading to potential ‘broad susceptibility’ (Chaudhary, 2013).

The brown planthopper (BPH), *Nilaparvata lugens* Stål (Homoptera: Delphacidae), is a monophagous herbivore of rice and causes heavy economic losses throughout Asia. These insects feed mainly on stems, and account for 28% of total plant dry matter reduction (Sōgawa, 1994). Feeding by low-density BPH populations can reduce rice yields (Watanabe *et al.*, 1997), while heavy infestation causes ‘hopperburn’ (Sōgawa, 1982). The BPH is also a vector of viruses responsible for ragged stunt and grassy stunt diseases. Control of BPHs using chemical pesticides is expensive, harmful to natural predators, and conducive to resistance build up in pests (Tanaka *et al.*, 2000). As such, development of insect-resistant crop varieties is of interest as an economical, environmentally friendly alternative strategy (Khush, 2001).

Beginning with the study of Pathak *et al.* (1969), considerable effort has been expended in the search for rice host resistance to BPHs. To date, 28 BPH resistance genes have been detected (Wu *et al.*, 2014) and mapped to six of the 12 rice chromosomes (2, 3, 4, 6, 11, and 12) (Cheng *et al.*, 2013). Among them, only *Bph14* has been cloned (Du *et al.*, 2009). Resistant rice varieties inhibit BPH oviposition and lower nymph survival rates, with little or no physiological stress or ensuing yield loss (Alam and Cohen, 1998*b*; Alagar *et al.*, 2007). Additional BPH resistance resources are consequently needed to support sustained control of these pests. Furthermore, studies of novel BPH resistance genes may provide more details of the molecular mechanisms underlying plant resistance to these insects.

In response to insect predation, plants have evolved a sophisticated system of defence mechanisms to deter herbivores. Two layers of the plant immune system perceive various invaders through different classes of immune receptors. Resistance (R) proteins which function in the second layer can effectively recognize specific pathogens that break through the first layer, and activate effector-triggered immunity (ETI) (Jones and Dangl, 2006). Most *R* genes which have been identified encode polymorphic ‘nucleotide-binding site plus leucine-rich repeat’ (NB-LRR) domains (Dangl and Jones, 2001). In addition, plant innate immunity involves the activation of expression changes to hormones such as abscisic acid, jasmonic acid (JA), salicylic acid (SA), and ethylene that mediate signalling cross-talk (Thompson and Goggin, 2006; Chen and Ronald, 2011; Consales *et al.*, 2012) and play a role in defence regulation against different pathogens (Thomma *et al.*, 1998). Pathogen response is usually regulated by an SA-dependent pathway and is associated with systemic acquired resistance (SAR), whereas the wounding response is usually controlled by a JA/ethylene-dependent pathway (Felton and Korth, 2000).

Previously, two BPH resistance genes, *bph20(t*) and *bph21(t*), were identified in RBPH54, a rice introgression line derived from wild rice *Oryza rufipogon* (Yang *et al.*, 2011). Genetic segregation in the F_2_ generation showed a ratio of 1:15, implying that the resistance was governed by recessive alleles at two loci. Examination of the BC_1_ generation also confirmed the duplicate interaction between *bph20(t*) and *bph21(t*). *bph20(t*) was mapped on the short arm of chromosome 6, and *bph21(t*) on the short arm of chromosome 10. The gene designations, *bph20(t*) and *bph21(t*), conflict with those of Rahman *et al.* (2009), who used the same names for genes that mapped to definitely different loci. To avoid confusion, the gene names *BPH29* and *BPH30* are proposed as replacements for *bph20(t*) and *bph21(t*), respectively, in accordance with the new CGSNL nomenclature system for rice (McCouch, 2008).

In this study, it was sought to fine-map further and clone *BPH29* using a map-based cloning approach. Seedling tests were also used to confirm the crucial function of *BPH29* in RBPH54 resistance to BPH. *BPH29* encodes a B3 DNA-binding domain-containing protein and is specifically restricted to vascular tissue, the site of BPH predation. In a BPH infestation experiment, RBPH54 defence responses included activation of the SA signalling pathway and suppression of the JA/ethylene-dependent pathway. The results provide a BPH resistance gene resource and offer valuable information regarding plant responses to herbivore pressure.

## Materials and methods

### Plant materials and growth conditions

Three rice sources, RBPH54, Taichuang Native 1 (TN1), and TR539, were used in this study. RBPH54 is an *indica* introgression line with BPH resistance derived from the wild rice species *O. rufipogon* Griff.; it has proved to be highly resistant to BPH biotypes 1 and 2 and resistant to biotype Bangladesh (Yang *et al.*, 2011). TN1, used as a control, is an international BPH-susceptible *indica* variety. TR539, containing *BPH29*, *BPH30*, and a few non-target background introgressions, is a line selected from a BC_3_F_2_ population constructed using TN1 as the recurrent parent and RBPH54 as the donor parent. All rice materials were grown in a greenhouse at 28 °C under 14h light/10h dark conditions or in test fields in Shanghai (31°11′N, 121°29′E) and Sanya (18°14′N, 109°31′E), China.

### Mapping population development and marker design

Near isogenic lines (NILs) were constructed by backcrossing TR539 to TN1 for two generations, followed by selfing to eliminate non-target introgressed genomic regions. From these NILs, one NIL heterozygous for the target locus *BPH29* (identified by markers BYL7 and BYL8), and recessive homozygous for *BPH30* (identified by markers RM222 and RM244) was selected and selfed to generate an NIL-F_2_ segregating population. NIL-F_2_ progeny were screened using insertion deletion (InDel) markers between BYL7 and BYL8, and recombinant plants were selected for further fine mapping of *BPH29*. Recombinant lines were evaluated for BPH resistance at the seedling stage by infestation with BPH biotype 2.

InDel markers for mapping were designed on the basis of sequence differences between *Japonica* rice Nipponbare (http://rgp.dna.affrc.go.jp/) and *indica* cultivar 9311 (http://rise.genomics.org.cn), using the online primer design tool Primer3 version 4.0 (http://primer3.ut.ee/).

### Gene transformation in rice

The binary plasmid vector pCAMBIA1304 (Center for the Application of Molecular Biology to International Agriculture) was assembled for the gene transformation. The pCAMBIA1304 vector carried kanamycin and hygromycin resistance for bacterial and transformed plant selection, respectively. Dehusked seeds of rice (RBPH54) were surface-sterilized by soaking in 70% (v/v) ethanol for 10min followed by 0.1% (w/v) HgCl_2_ for 20min, washed with sterile distilled water, and sown on gel medium for embryogenic callus induction. After bombardment with a gene gun, calli were selected after a 2 week exposure to 50mg l^−1^ hygromycin. Hygromycin-resistant calli regenerated and grew into transformed T_0_ lines. For each gene transformation, 10–20 independent T_0_ lines were collected. Genomic DNAs were extracted from these transformed lines as templates for PCR, and the hygromycin gene was then amplified to confirm the transformation.

### BPH resistance bioassay

BPHs were collected from fields in Nanning (22°48′N, 108°22′E) and females were reared separately. F_1_ lines were evaluated for BPH biotype. Insects of BPH biotype 2 were selected and maintained on TN1 under greenhouse conditions (25–30 °C) at the Plant Protection Research Institute, Guangxi Academy of Agricultural Sciences.

The BPH resistance of rice plants was evaluated by the modified standard seed-box screening technique (Brookes and Barfoot, 2003). About 20 seeds of each rice line were sown in a row in a metal box (80×55×8cm) along with resistant control RBPH54 and susceptible control TN1. When seedlings reached the three-leaf stage, they were infested with first- to second-instar BPHs (biotype 2) at a density of 10 insects per seedling. When all the TN1 seedlings had died, damage to seedlings was scored as 0, 1, 3, 5, 7, or 9 according to International Rice Research Institute (IRRI) guidelines (IRRI, 1988). Lower scores indicate higher resistance to BPHs.

### RNA isolation and quantitative real-time PCR (qRT-PCR)

Total RNAs of different rice lines and tissues were isolated using an RNAprep plant kit (Tiangen, Beijing, China). First-strand cDNA was generated using a Primescript RT reagent kit (Perfect Real Time; Takara, Otsu, Shiga, Japan). qRT-PCR analysis was carried out using the SYBR *Premix Ex Taq* (Takara) on a CFX96 Real-Time system (Bio-Rad). Samples were amplified by two-step qRT-PCR, which was 40 cycles of 95 °C for 5 s and 60 °C for 30 s. Melting curves and standard curves were calculated and analysed, with relative mRNA expression levels normalized using *OsActin1* as a reference. Primers used in this study are shown in Supplementary Table S2 available at *JXB* online.

### Subcellular localization

The full-length coding region of *BPH29* was cloned from RBPH54 cDNA using *G5* Vector primer (Supplementary Table S2 at *JXB* online), assembled into the vector pCAMBIA1304, and fused in-frame with green fluorescent protein (GFP) under the control of the *Cauliflower mosaic virus* (CaMV) 35S promoter. The modified pCAMBIA1304 construct and control were transiently transformed into rice protoplasts. The rice protoplasts were prepared from three-leaf stage plant leaves and transformed using 40% polyethylene glycol solution (Yoo *et al.*, 2007). After 16–24h incubation at 28 °C in darkness, the protoplast location of the fusion protein was observed by confocal fluorescence microscopy (LSM A710; Zeiss).

### β-Glucuronidase expression analysis

A 1.5kb 5′-upstream (−1500 to −1 for *BPH29*-ATG and 29 818 to 31 317 for P0514G12) segment of the *BPH29* promoter region was amplified with the *G5* promoter primer (Supplementary Table S2 at *JXB* online) and cloned into a promoterless pCAMBIA1301 vector followed by the β-glucuronidase (*GUS*) reporter gene. This P_*BPH29*_::*GUS* fusion vector was introduced into RBPH54, and 30–40 independent positive T_0_ lines were collected for subsequent experiments. *GUS* activity was detected by histochemical GUS staining (Jefferson *et al.*, 1987) and observed by light microscopy.

### Hormone measurements

About 40 seeds of each rice line were sown in soil and grown to the three-leaf stage, and then the seedlings were infected with first- to second-instar BPHs (biotype 2) at a density of 10 insects per seedling. Rice leaves were harvested 0, 24, and 48h after BPH infestation, frozen in liquid nitrogen, and ground to a fine powder for hormone measurements. Quantification of endogenous SA and JA was performed as described in Chen *et al.* (2012).

### Accession numbers

Sequence data from this article can be found in the EMBL/Gen Bank data libraries under accession numbers: LOC_Os06g01820, LOC_Os06g01830, LOC_Os06g01840, LOC_Os06g01850, LOC_Os06g01860, LOC_Os06g01870, KC019172, KC019173, Os03g0718100, D38170, X87946, X89859, AY923983, AY062258, D14000, and AY396568.

## Results

### Fine mapping of *BPH29*

**Fig. 1.**
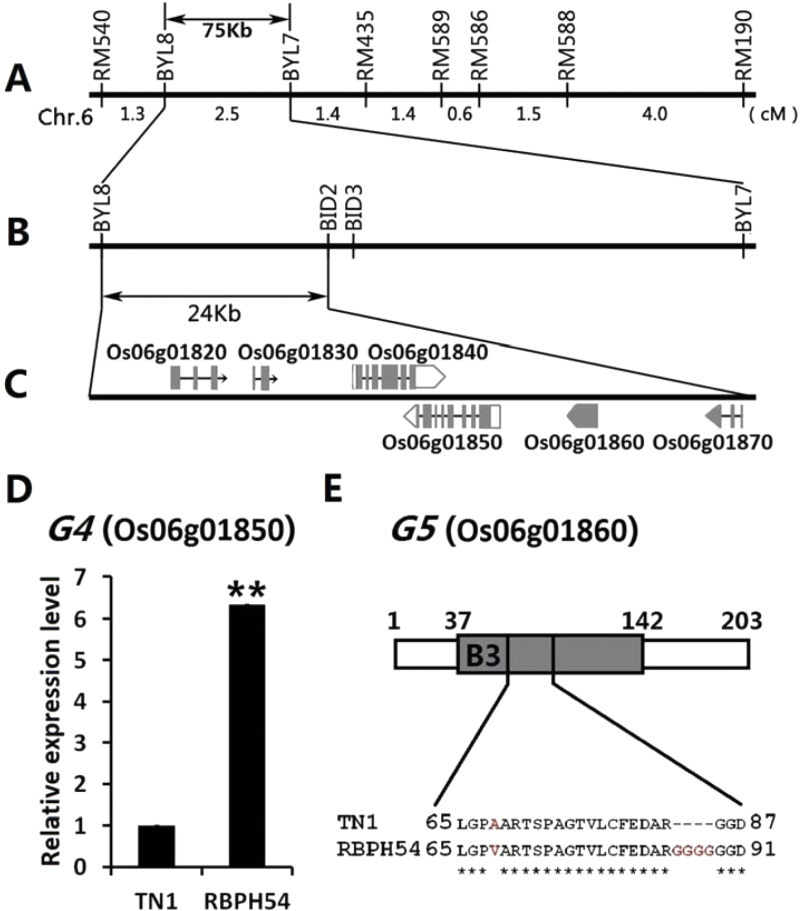
Fine mapping and prediction of the *BPH29* gene. (A) *BPH29* location in a 75kb region between BYL8 and BYL7 on the short arm of chromosome 6 as determined previously. (B) Further refinement of the locus region to a 24kb location between BYL8 and BID2. (C) The six predicted genes (LOC_Os06g01820, LOC_Os06g01830, LOC_Os06g01840, LOC_Os06g01850, LOC_Os06g01860, and LOC_Os06g01870) annotated in this region. (D) Quantitative real-time PCR (qRT-PCR) analysis of *G4* (LOC_Os06g01850) expression in rice lines Taichuang Native 1 (TN1) and RBPH54. Student’s *t*-test: **P<0.01. Data are means ±SD (*n*=3 individuals), and expression levels are shown relative to that of *OsActin1*. (E) Protein structure of *G5* (LOC_Os06g01860) annotated by Pfam (http://pfam.xfam.org/). The B3 DNA-binding domain is indicated by a grey box. Differences in *G5* between TN1 and RBPH54 amino acid sequences include a single mutation and a four-glycine insertion. Identical amino acids are marked with a single asterisk.

To narrow down the *BPH29* locus region, a NIL-F_2_ segregating population which is heterozygous for the target locus *BPH29* and recessive homozygous for *BPH30* was developed. A total of 3435 NIL-F_2_ progeny were subjected to InDel analysis with markers between BYL7 and BYL8 (Fig. 1A). Out of five InDel markers, BID2 and BID3 exhibited polymorphism between the introgression line parents. As a result, 32 recombinants were identified between BYL8 and BID2; 18 of these restricted *BPH29* to the region downstream of BYL8, while the remaining 14 localized the gene to the region upstream of BID2 (Table 1). For each of the 32 recombinant families, 20 homozygous selfed progeny (F_2:3_ family) were genotyped by the appropriate segregating markers and analysed for resistance to BPH. BLAST analysis revealed that a PAC (P1-derived artificial chromosome) clone, P0514G12, was anchored by BYL8 and BID2. In this fashion, *BPH29* was confined to a 24kb region flanked by BYL8 and BID2 (Fig. 1B).

**Table 1.** Genotypes of key recombinants from the NIL-F_2_ population

Flanked marking	Individual^*a*^	Chromosome	Markers^*b*^			
			RM540	BYL8	BID2	BYL7
BYL8	BPH-132	6	A	A	B	B
	BPH-133	6	A	A	B	B
	BPH-134	6	A	A	B	B
	BPH-135	6	A	A	B	B
	BPH-136	6	A	A	B	B
	BPH-138	6	A	A	B	B
	BPH-139	6	A	A	B	B
	BPH-140	6	A	A	B	B
	BPH-141	6	A	A	B	B
	BPH-142	6	A	A	B	B
	BPH-416	6	A	A	B	B
	BPH-417	6	A	A	B	B
	BPH-419	6	A	A	B	B
	BPH-1097	6	A	A	B	B
	BPH-1100	6	A	A	B	B
	BPH-1103	6	A	A	B	B
	BPH-1104	6	A	A	B	B
	BPH-1106	6	H	H	B	B
BID2	BPH-49	6	B	B	H	H
	BPH-91	6	B	B	A	A
	BPH-110	6	B	B	A	A
	BPH-143	6	B	B	H	H
	BPH-151	6	B	B	H	H
	BPH-159	6	B	B	H	H
	BPH-162	6	B	B	H	H
	BPH-172	6	B	B	H	H
	BPH-183	6	B	B	H	H
	BPH-211	6	B	B	H	H
	BPH-246	6	B	B	H	H
	BPH-251	6	B	B	A	A
	BPH-254	6	B	B	A	A
	BPH-259	6	B	B	H	H

^*a*^ For each of the 32 recombinant families, 20 homozygous selfed progeny (F_2:3_ family) were evaluated for BPH resistance at the seedling stage by infestation with BPH biotype 2 and identified as resistant to BPH.

^*b*^ A, TN1 homozygous genotype; B, RBPH54 homozygous genotype; H, heterozygous genotype.

### Analysis of candidate genes

Six genes (LOC_Os06g01820, LOC_Os06g01830, LOC_Os06g01840, LOC_Os06g01850, LOC_Os06g01860, and LOC_Os06g01870) were predicted in the 24kb target region based on annotations in the TIGR Rice Genome Annotation Project database (http://rice.plantbiology.msu.edu/) (Kawahara *et al.*, 2013) (Fig. 1C). LOC_Os06g01850 and LOC_Os06g01860 showed functional differences between the susceptible parent TN1 and the resistant parent RBPH54, and were designated as *G4* and *G5*, respectively.

An 11bp insertion was present in the 505bp upstream region of the *G4* start codon of RBPH54 compared with TN1. This regulatory region difference may have an impact on *G4* expression levels, and remarkably high levels of *G4* expression were detected in RBPH54 by qRT-PCR (Fig. 1D). Differences of *G5* were found in its coding regions. These differences included three single nucleotide polymorphism, resulting in one single amino acid mutation from alanine to valine, and a 12bp insertion corresponding to four glycines in RBPH54 (Fig. 1E; Supplementary Fig. S1 at *JXB* online). These *G5* sequence discrepancies between the two mapping parents may affect the structure and function of the encoded protein. On the basis of these observations, *G4* and *G5* were selected as candidates for the BPH resistance gene *BPH29*.

### Complementation tests

**Fig. 2.**
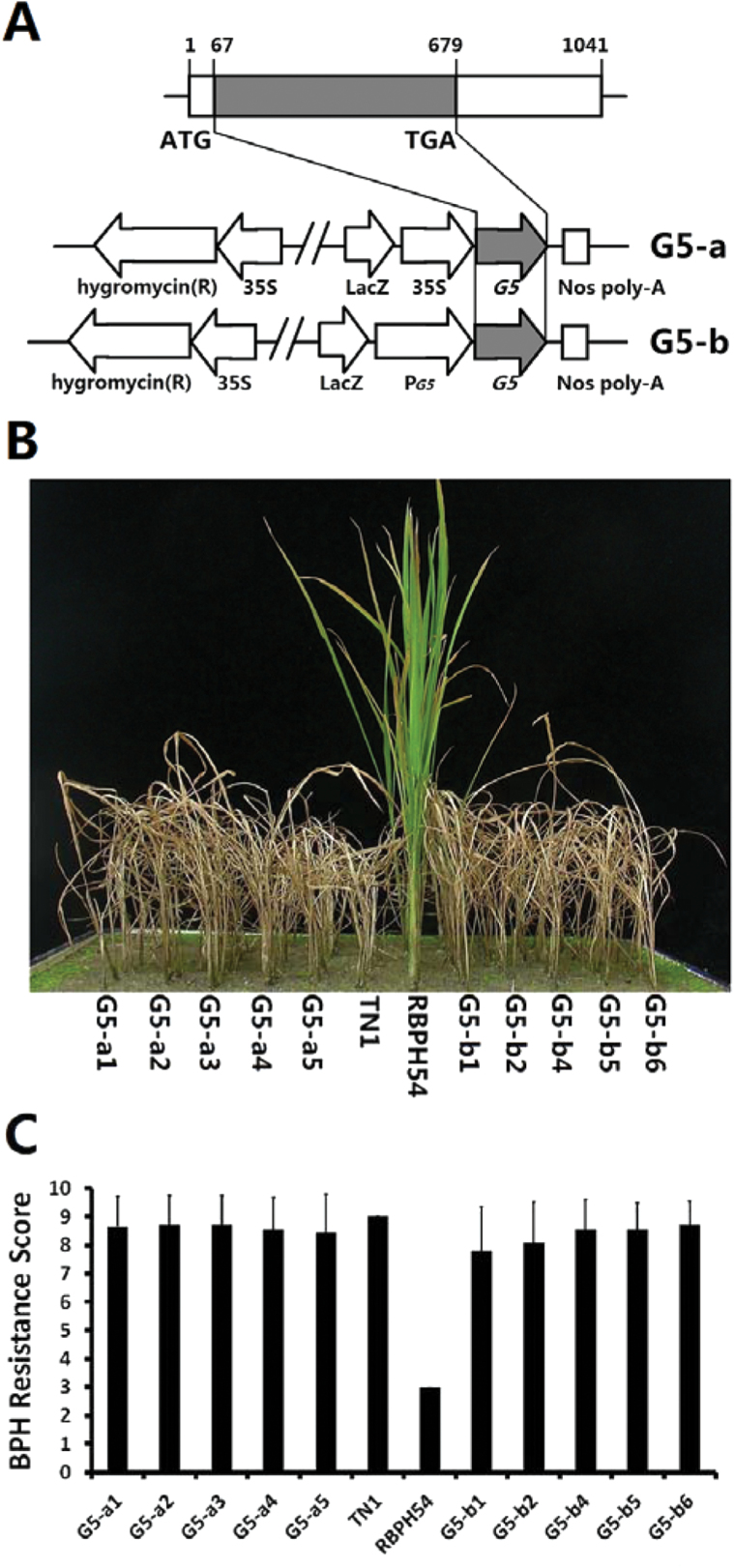
Complementation test for *BPH29*. (A) Gene and vector structure of *G5*. *G5* has only one exon, with the open reading frame indicated by a grey box. Two linear maps of pCAMBIA1304 are shown: G5-a and G5-b represent the vector assembled with the 35S and *G5* native promoter, respectively. (B) BPH resistance bioassay of *G5* transgenic lines at the seedling stage. G5-a1 to G5-a5, 35S::*G5* transgenic T_1_ lines; G5-b1 to G5-b6, P_*G5*_::*G5* transgenic T_1_ lines; TN1, susceptible parent control; RBPH54, resistant parent control. (C) Brown planthopper (BPH) resistance scores of *G5* transgenic lines. Both G5-a and G5-b transgenic lines showed higher scores, indicating their high susceptibility to BPH. Data are means ±SD (*n*=40 plants).

To investigate which candidate gene corresponded to *BPH29*, complementation tests were performed. Different strategies were adopted based on discrepancies in *G4* and *G5*. An antisense transformation experiment was conducted to repress the expression of endogenous *G4* in RBHP54, and the dominant *G5* allele was transferred from TN1 into RBPH54 to mask the phenotype of the recessive *G5* allele. An antisense-*G4* vector was generated by fusion of the CaMV 35S promoter with the antisense *G4* coding sequence (Supplementary Fig. S2A at *JXB* online). For *G5*, the dominant allele was cloned from TN1 and assembled downstream of either the CaMV 35S promoter (35S::*G5*) or the *G5* 1.5kb (−1500 to −1 for *G5*-ATG, and 29 818 to 31 317 for P0514G12) native promoter (P_*G5*_::*G5*) (Fig. 2A). These transformations were all performed in the resistant parent RBPH54 using a pCAMBIA1304 vector.

After seedling tests in the transgenic population, it was found that loss of BPH resistance in RBPH54 co-segregated with the *G5* dominant allele gene transformations. In the BPH resistance bioassay, both 35S::*G5* and P_*G5*_::*G5* transformation lines showed high susceptibility to BPH, while RBPH54 seedlings remained healthy (Fig 2B, C). cDNA sequencing confirmed that the *G5* dominant allele had been expressed successfully in these BPH-susceptible transformation lines (Supplementary Fig. S3 at *JXB* online). The 35S::anti-*G4* positive transformants were still highly resistant to BPH (Supplementary Fig. S2B, C, D). It was therefore concluded that *G5* was *BPH29*. Scores of *G5* transgenic lines in the bulked seedling test revealed a significant loss of resistance, quite similar to levels of the susceptible control TN1 (Fig. 2C), indicating that the recessive allele of *BPH29* is crucial to the BPH resistance of RBPH54. Sequence data of both dominant and recessive alleles have been deposited in GenBank under accession numbers KC019172 and KC019173, respectively.

### Analysis of BPH29 protein

**Fig. 3.**
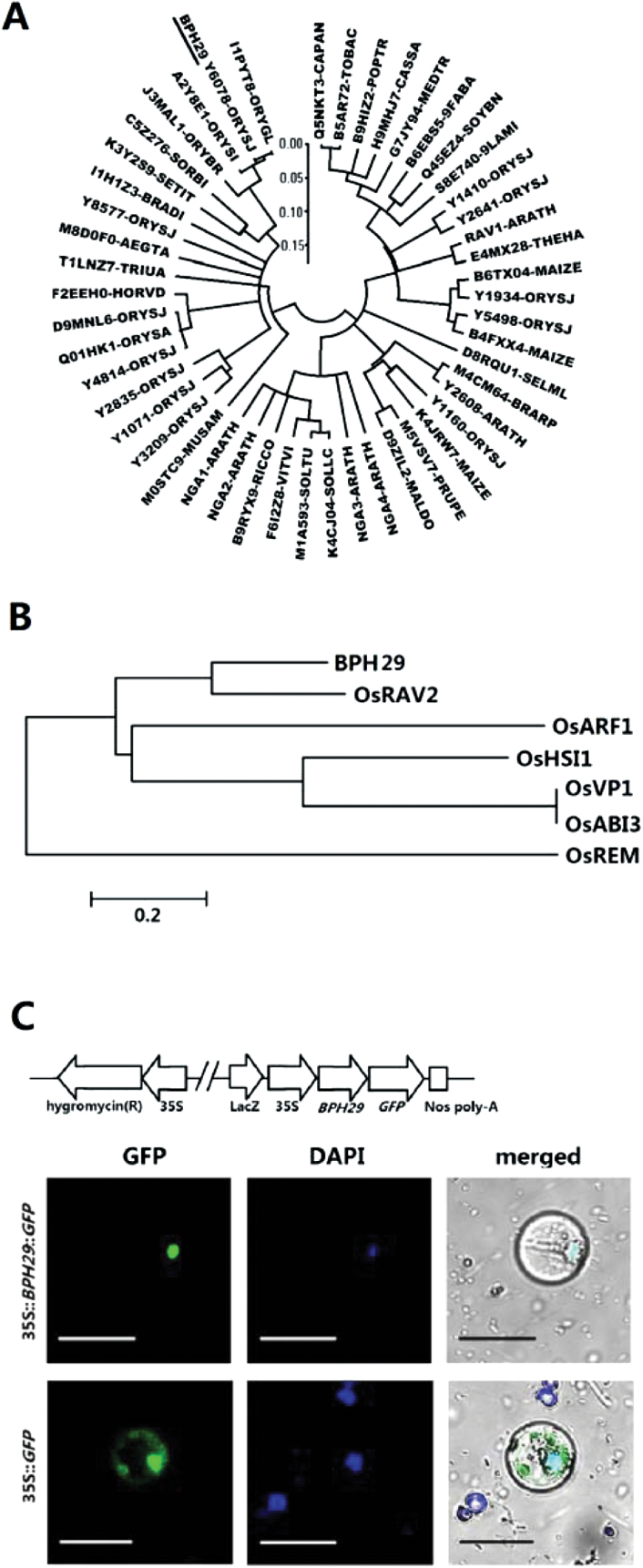
Analysis of BPH29 protein. (A) Amino acid sequence homologue analysis of BPH29. BPH29 homologues are extensively distributed among higher plants, such as rice, *Arabidopsis*, tomato, pepper, soybean, potato, wheat, maize, sorghum, and tobacco. Analysis was run by UniProt (http://www.uniprot.org/). The tree was generated by the Neighbor–Joining method (scale bar=0.05 estimated amino acid substitutions per residue). (B) Homology analysis of the *BPH29* B3 domain. B3 domains of *BPH29*, *OsABI3* (NM_001051697), *OsVP1* (D16640), *OsRAV2* (AK241984), *OsARF1* (AK065936), *OsHSI1* (AK241199), and *OsREM* (NM_001185617) were analysed by MEGA 4. The tree was generated by the Neighbor–Joining method (scale bar=0.2 estimated amino acid substitutions per residue). (C) Subcellular localization of *BPH29*. The 35S::*BPH29*::*GFP* fusion protein was transiently transformed into rice protoplasts. Green fluorescent protein (GFP) fluorescence was detected co-localized with 4′,6-diamidino-2-phenylindole (DAPI), a nucleus-specific fluorescent stain, in the nucleus. GFP fluorescence in control protoplasts (35S::*GFP*) was detected throughout the cytosol. Scale bar=30 μm.

Amino acid sequence alignment analysis showed that BPH29 homologues are extensively distributed among various species of higher plants, such as rice, *Arabidopsis*, tomato, pepper, soybean, potato, wheat, maize, sorghum, and tobacco (Fig. 3A). BPH29 in *Oryza sativa* shares 96% and 77% sequence identity with its homologues in *Oryza glaberrima* and *Oryza brachyantha*, respectively. The widespread presence and high similarity of BPH29 reveals that the *BPH29* gene is highly conserved in the plant kingdom and may have a critical function.

According to information in GenBank, *BPH29* is a single-copy gene that encodes a 203 amino acid (recessive allele) putative uncharacterized protein with no introns. Amino acid sequence analysis has shown that BPH29 contains only one B3 DNA-binding domain (Pfam accession 02362) (Punta *et al.*, 2012) (Fig. 1E), a highly conserved domain found exclusively in transcription factors that interacts with the major groove of DNA (Yamasaki *et al.*, 2004). Considering this characteristic, BPH29 should be localized to the nucleus. To confirm the subcellular localization of the BPH29 protein, the *BPH29* coding region was fused to the *GFP* gene under the control of the 35S promoter (35S::*BPH29*::*GFP*). After the activity was confirmed by gene transformation and BPH resistance bioassay in rice (Supplementary Fig. S4 at *JXB* online), this BPH29–GFP fusion protein was transiently transformed into rice protoplasts, and its fluorescence was detected in the nucleus by co-localization with nuclear-specific 4′,6-diamidino-2-phenylindole (DAPI) (Fig. 3C). This result indicates that BPH29 is a nuclear-localized protein.

### Expression analysis of *BPH29*

**Fig. 4.**
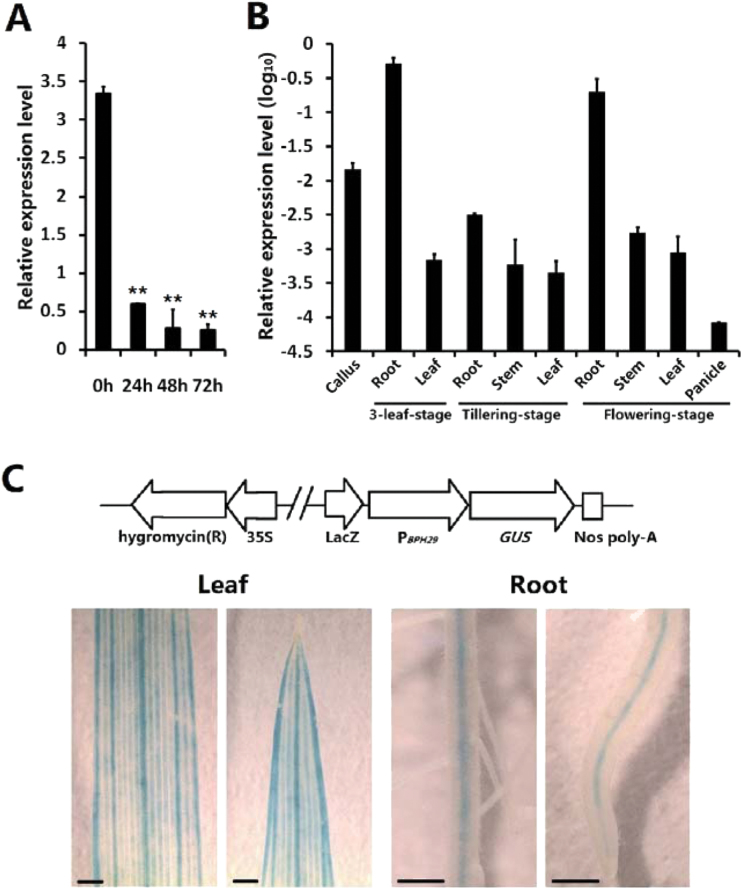
Expression analysis of *BPH29*. (A) qRT-PCR analysis of the *BPH29* expression pattern under BPH infestation. RBPH54 plants were collected 0, 24, 48, and 72h after infestation. Expression levels revealed that *BPH29* is suppressed by BPH infestation. Student’s *t*-test: **P<0.01. Data are means ±SD (*n*=3 individuals). Expression levels are relative to *OsActin1*. (B) qRT-PCR analysis of *BPH29* spatial- and temporal-specific expression. Different tissues from RBPH54 callus, three-leaf, tillering, and flowering stages were detected. *BPH29* showed relatively high levels in callus and in roots at the three-leaf and flowering stages. Data are means ±SD (*n* = 3 individuals). Expression levels are relative to *OsActin1* and log_10_ transformed. (C) Histochemical localization of *BPH29* revealed by β-glucuronidase (*GUS*). The *BPH29* promoter was fused with the *GUS* reporter gene (P_*BPH29*_::*GUS*), and introduced into RBPH54. *GUS* activity is indicated in blue. It showed that *GUS* stained the vascular system of leaf and root. Scale bar=1mm.

Expression level changes in *BPH29* following the BPH infestation were detected. RBPH54 seedlings on the three-leaf stage were infested with first- to second-instar BPHs, and whole plants were harvested 0, 24, 48, and 72h later. The qRT-PCR analysis revealed that *BPH29* decreased rapidly 24h after BPH infestation, and remained low afterwards (Fig. 4A). This expression pattern suggests that *BPH29* is suppressed following BPH infestation.

Based on the CREP rice gene expression database (http://crep.ncpgr.cn/), *BPH29* showed extremely low expression levels in most tissues throughout the entire rice life cycle, except for roots of seedlings with two tillers (Supplementary Table S1 at *JXB* online). A qRT-PCR analysis was carried out to confirm this expression pattern using various RBPH54 tissue materials obtained from callus, three-leaf, tillering, and flowering stages. The qRT-PCR analysis confirmed that *BPH29* was expressed at low basal levels, with marked increases during the callus stage and in roots at the three-leaf and flowering stages (Fig. 4B). These plant parts are located near the region of ingestion during BPH predation on rice plants.

To characterize tissue specificity of *BPH29* expression, *GUS* histochemical localization of the *BPH29* gene was examined. The *BPH29* promoter (−1500 to −1 for *BPH29*-ATG) was fused to the *GUS* reporter gene and introduced into RBPH54. GUS activity was strongly detected in the vascular system of leaf and root (Fig. 4C), where BPH sucked the sap by using its stylet. Above all, this specific expression pattern of *BPH29* is consistent with the location of BPH attack.

### Defence-related gene and hormone modulation in *BPH29*-mediated insect resistance

Plant responses to herbivores involve global changes in gene expression mediated by multiple signalling pathways, including plant hormone SA and JA/ethylene signalling pathways (Walling, 2000). BPH infestation experiments were performed on three-leaf stage rice to investigate the changes of defence-related genes transcript level as well as the corresponding hormones level during infestation (Qiu *et al.*, 2007) and to compare differences between BPH-resistant RBPH54 and loss-of-resistance *G5* transformation lines.

**Fig. 5.**
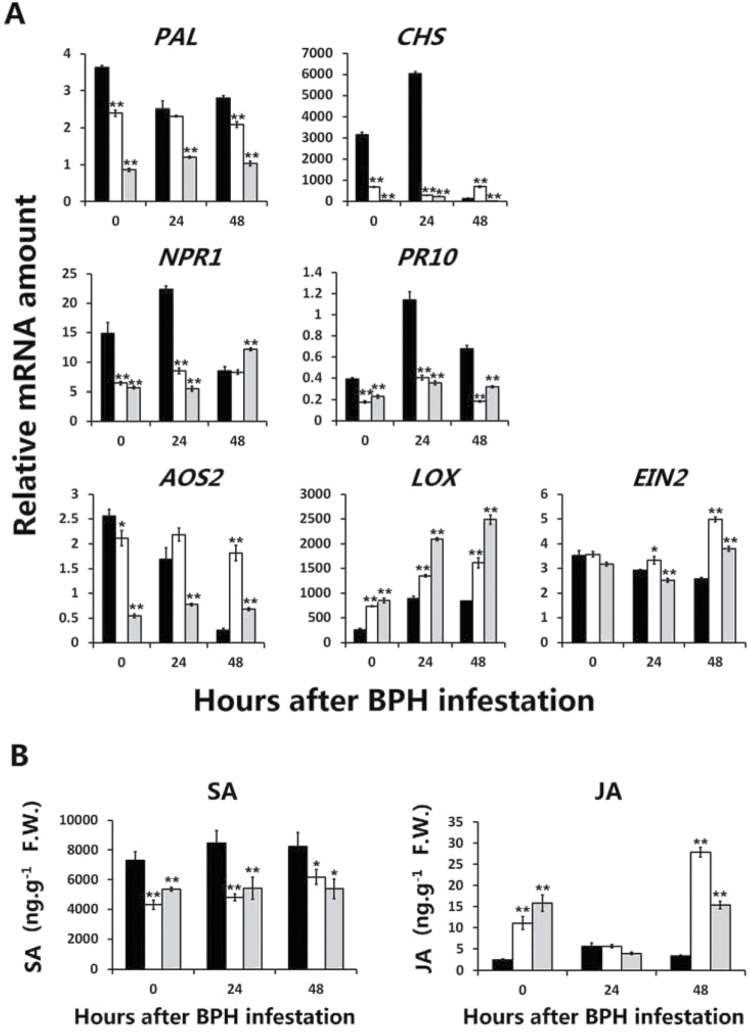
Quantification of plant defence-related genes and hormone amounts in *BPH29*-mediated insect resistance. (A) Expression analysis of plant defence-related genes. *PAL* and *CHS* are two salicylic acid (SA) synthesis-related genes in the phenylpropanoid SA synthesis pathway. *NPR1* is a key regulator of systemic acquired resistance (SAR) and operates downstream of the SA pathway. *PR10* is one of the pathogenesis-related (*PR*) genes. *AOS2* and *LOX* are two important genes in the jasmonic acid (JA) synthesis pathway. *EIN2* is the ethylene signalling pathway receptor gene. *OsActin1* was used as a reference control. (B) Hormone measurements of SA and JA levels. For (A) and (B), black bars represent BPH-resistant RBPH54, white bars correspond to the BPH-susceptible transgenic line G5-a1, and grey bars to G5-a2. Samples were obtained 24h and 48h after BPH infestation; 0h corresponds to no insect treatment. Student’s *t*-test: *P<0.05; **P<0.01. Data are means ±SD (*n*=3).

SA synthesis can be accomplished via either the isochorismate or phenylpropanoid pathways (Lee *et al.*, 1995; Mauch *et al.*, 2001). Two genes in the phenylpropanoid pathway, *PAL* (phenylalanine ammonia-lyase) and *CHS* (chalcone synthase), showed significant high transcript levels in RBPH54. *CHS*, in particular, showed pronounced background transcription in RBPH54 and rapid accumulation 24h after infestation. *NPR1* (homologue of *Arabidopsis* non-expressor of pathogenesis-related genes 1), a key regulator of SAR in *Arabidopsis* that functions downstream of SA signalling pathways (Durrant and Dong, 2004), was transcribed at high levels in RBPH54. One of the *PR* (pathogenesis-related) genes *PR10* was also higher in RBPH54 than in *G5* lines (Fig. 5A). LOX and AOS are two important enzymes in the JA synthesis pathway (Zhao *et al.*, 2005). After BPH infestation, *AOS2* (allene oxide synthase 2) decreased rapidly in RBPH54, with no significant change observed in BPH-susceptible *G5* lines. *LOX* (lipoxygenase) transcript levels were generally lower in RBPH54 as well. Additionally, expression of the ethylene signalling pathway receptor gene *EIN2* (ethylene insensitive 2) (Jun *et al.*, 2004) was higher in loss-of-resistance *G5* lines than in RBPH54 at 48h after infestation (Fig. 5A).

Quantification results of hormone amounts were consistent with the gene transcript levels above (Fig. 5B). The amount of SA showed a higher level in RBPH54, and quantification of JA confirmed the lower JA level in RBPH54. These results indicate that BPH-resistant RBPH54 activate SA synthesis after BPH feeding, and may defend against insects through SA-dependent SAR. In the meanwhile, the JA/ethylene-dependent pathway was suppressed during RBPH54 defence against BPH feeding.

## Discussion

### *BPH29*, a new recessive BPH resistance gene

Owing to climate change, pest frequency has become a critical problem that threatens global food security. To date, 28 BPH resistance genes have been detected, and only one (*Bph14*) has been cloned and published (Du *et al.*, 2009). In this study, the *BPH29* locus region was narrowed down to 24kb on the short arm of chromosome 6, and the location of *BPH29* was finally confirmed by genetic analysis in this target region (Figs 1, 2; Supplementary Fig. S2 at *JXB* online). Thus a new BPH resistance gene, *BPH29*, has been cloned.

BPH resistance is known to be monogenically controlled in most resistant sources, and little progress has been made on recessive resistance genes compared with dominant ones (Huang *et al.*, 2013). RBPH54 exhibits sustainable resistance to BPHs that is governed by recessive alleles at two loci (Yang *et al.*, 2011). Generally, mechanisms of plant resistance to insects can be categorized into antixenosis, antibiosis, and tolerance (Painter, 1951). Antixenosis refers to a quality that repels or disturbs insects (Alam and Cohen, 1998*a*). In the present study, the recessive *BPH29* allele that contains a DNA mutation in the B3 domain (Fig. 1E; Supplementary Fig. S1 at *JXB* online) might lose the function of a dominant allele which was required for the settling of insects, and confer an antixenosis resistance in conjunction with another recessive locus. Ultimately, formal proof of the role of *BPH29* in antixenosis resistance should be tested in the future, and the mechanism of co-operation of the two recessive loci remains to be explored. Cloning of *BPH29* provides valuable information on molecular mechanisms of recessive resistance genes and offers a chance to understand the uncommon resistance sources conferred by polygenes.

The interaction between rice and the BPH reflects the co-evolutionary arms race between plants and herbivorous pests (Cheng *et al.*, 2013) and provides an ideal system for studying the molecular mechanisms underlying plant defences against phloem-feeding insects, which are still fairly unclear. Further efforts related to the identification of herbivore-specific signal molecules, their recognition, and signal transduction might result in major breakthroughs in the future (Zebelo and Maffei, 2015). The information gleaned from *BPH29* offers further insights into the field of plant–insect interactions and plant defence response.

### *BPH29* encodes a B3 domain-containing resistance protein

An essential layer of the plant immune system is based on highly polymorphic R proteins and is effective against specialized pathogens (Jones and Dangl, 2006). Most R proteins are multidomain NB-LRR proteins (Takken and Tameling, 2009). *Mi-1.2*, *Vat*, and *Bph14* (Rossi *et al.*, 1998; Pauquet *et al.*, 2004; Du *et al.*, 2009), the three plant insect resistance genes that have been cloned, all encode NB-LRR R proteins (Bruce, 2015). *BPH29* is a single-exon gene that encodes a 203 amino acid protein which only contains one B3 DNA-binding domain (Fig. 1E), that is a novel structure for the *R* proteins.

The B3 domain is a highly conserved domain found only in vascular plants (Woo *et al.*, 2010). Five major gene classes containing the B3 domain have been identified: ABI3/VP1 (*Abscisic acid insensitive3*/*Viviparous1*) (Giraudat *et al.*, 1992; Suzuki *et al.*, 1997), HSI (high-level expression of sugar-inducible gene) (Tsukagoshi *et al.*, 2005), RAV (related to ABI3/VP1) (Kagaya *et al.*, 1999), ARF (auxin response factor) (Ulmasov *et al.*, 1997), and REM (reproductive meristem) (Franco-Zorrilla *et al.*, 2002) gene families. Among these genes, the B3 domain of *BPH29* is most similar to that of the RAV family (Fig. 3B). With regard to function, genes from the different subfamilies are involved in similar issues, such as hormone signalling pathways, flowering time control, organ growth, and polarity (Swaminathan *et al.*, 2008). A previous study has shown that the *RAV1* gene plays an important role in bacterial disease resistance (Sohn *et al.*, 2006). Identification of *BPH29* offers a unique example of the B3 domain’s role in plant insect resistance function.

### Similarity of plant BPH resistance responses mediated by *BPH29* and plant defences against pathogens

Molecular responses of plants against herbivores are mainly correlated with insect feeding modes and degree of plant tissue damage. In contrast to chewing insects that cause extensive damage to plant foliage and activate wound response pathways, BPHs are typical phloem-feeding insects that suck sap using their stylets, and therefore cause minimal physical injury to the host, but the interaction between insect stylets and plant cells is prolonged and intimate (Du *et al.*, 2009). In some respects, these features are similar to attacks arising from fungal pathogens (Jones and Dangl, 2006; Wang *et al.*, 2008). Additionally, BPHs also act as virus vectors, causing insect feeding to be accompanied by plant pathogen-related defences. As a consequence, the resistance factors are thought to occur within the phloem (Walling and Thompson, 2012), and host responses to phloem-feeding insects are thought to mirror responses to fungal or bacterial pathogens (Walling, 2000, 2008).

The present results revealed that the tissue-specific expression of *BPH29* was restricted to plant vascular tissue, the location of BPH attack (Fig. 4C). Plant hormone pathway responses to BPH suggest the activation of SA-dependent SAR, and the JA/ethylene-dependent pathway was suppressed during RBPH54 defence against BPHs (Fig. 5). These characteristics are consistent with molecular responses that occur during plant–pathogen interaction (Felton and Korth, 2000). These facts indicate that *BPH29*-mediated resistance against the BPH is similar to defensive molecular responses that plants apply to biotrophic pathogens.

### A valuable host resistance gene resource for crop breeding development

During crop breeding improvement, the elimination of insect damage to broadly susceptible domesticated modern crops is desired, with an economical and environmentally friendly strategy strongly preferred. Genetic resources from natural wild germplasm may be able to meet these demands (Bruce, 2012). Common cultivated rice possesses an AA genome. The host genetic background is an important factor that influences the function of resistance genes (Cao *et al.*, 2007). RBPH54 resistance is derived from the wild rice *O. rufipogon*, which has a close evolutionary relationship with *Oryza sativa* and possesses the same AA genome as Asian rice cultivars. This background suggests that *BPH29* is able to maintain a fine interaction with the cultivated rice genome. In addition, a single BPH resistance gene has reportedly been quickly overcome by insects under natural conditions (Khush and Brar, 1991), with the pyramiding of two or three genes generally found to provide greater resistance (Sharma *et al.*, 2004; Hu *et al.*, 2012). Considering the frequency of resistant BPH outbreaks and the need to pyramid multiple resistance genes for greater resistance, the identification of the BPH resistance gene *BPH29* should greatly facilitate the breeding of rice host-resistant varieties.

## Supplementary data

Supplementary data are available at *JXB* online.

Figure S1. Differences in *G5* between the susceptible parent TN1 and the resistant parent RBPH54.

Figure S2. Complementation test for *G4*.

Figure S3. Expression of *G5* alleles in transgenic lines.

Figure S4. BPH29–GFP fusion protein activity test.

Table S1. Transcript levels of *BPH29* and *OsActin1* at different rice developmental stages.

Table S2. Primers used in this work.

Supplementary Data
