# Corrigendum: Map-based cloning and characterization of *BPH29*, a B3 domain-containing recessive gene conferring brown planthopper resistance in rice

**DOI:** 10.1093/jxb/erx466

**Published:** 2018-01-24

**Authors:** Ying Wang, Liming Cao, Yuexiong Zhang, Changxiang Cao, Fang Liu, Fengkuan Huang, Yongfu Qiu, Rongbai Li, Xiaojin Luo

**Affiliations:** 1 State Key Laboratory of Genetic Engineering, Institute of Genetics, School of Life Sciences, Fudan University, Shanghai, China; 2 Crop Breeding and Cultivation Research Institute, Shanghai Academy of Agricultural Sciences, Shanghai, China; 3 State Key Laboratory for Conservation and Utilization of Subtropical Agro-bioresources and College of Agriculture, Guangxi University, Nanning, China; 4 Plant Protection Research Institute, Guangxi Academy of Agricultural Sciences, Nanning, China

*Journal of Experimental Botany,* Vol. 66, No. 19 pp. 6035–6045, 2017 doi: 10.1093/jxb/erv318

In the original version of this paper, a contributing author’s name was spelt incorrectly.

“Ying Wang, Liming Cao, Yuexiong Zhang, Changxiang Cao, Fang Liu, Fengkuan Huang, Yongfu Qiu, Rongbai Li and Xiaojin Lou”

The corrected list of authors now reads as follows:

“Ying Wang, Liming Cao, Yuexiong Zhang, Changxiang Cao, Fang Liu, Fengkuan Huang, Yongfu Qiu, Rongbai Li and Xiaojin Luo”

