# Supplementary material for: Map-based cloning and characterization of BPH29, a B3 domain-containing recessive gene conferring brown planthopper resistance in rice
Source: J Exp Bot. 2015 Jul 1;66(19):6035–45. doi: 10.1093/jxb/erv318 (PMC4566989; doi:10.1093/jxb/erv318)
Supplement: Supplementary Data [file supp_66_19_6035__index.html]

Map-based cloning and characterization of BPH29, a B3 domain-containing recessive gene conferring brown planthopper resistance in rice — Supplementary Data 

# Map-based cloning and characterization of *BPH29*, a B3 domain-containing recessive gene conferring brown planthopper resistance in rice

## Supplementary Data

Data files

- Supplementary Data - Supplementary Data
