# Supplementary material for: Map-based cloning and characterization of BPH29, a B3 domain-containing recessive gene conferring brown planthopper resistance in rice
Source: J Exp Bot. 2015 Jul 1;66(19):6035–45. doi: 10.1093/jxb/erv318 (PMC4566989; doi:10.1093/jxb/erv318)
Supplement: Supplementary Data [file supp_erv318_Supporting_data.pdf]

Title: Map-based cloning and characterization of *BPH29*, a B3 domain-containing recessive gene conferring brown planthopper resistance in rice

Authors: Ying Wang[1], Liming Cao[2], Yuexiong Zhang[3], Changxiang Cao[1], Fang Liu[3], Fengkuan Huang[4], Yongfu Qiu[3], Rongbai Li[3,*] and Xiaojin Lou[1,*]

Supplementary Data

Supplementary Fig. S1. Differences of *G5* between susceptible-parent TN1 and resistant-parent RBPH54.

Supplementary Fig. S2. Complementation test for *G4*.

Supplementary Fig. S3. Expression of *G5* alleles in transgenic lines.

Supplementary Fig. S4. BPH29-GFP fusion protein activity test.

Supplementary Table S1. Transcript levels of *BPH29* and *OsActin1* at different rice developmental stages

Supplementary Table S2. Primers used in this work

Supplemental Figures:

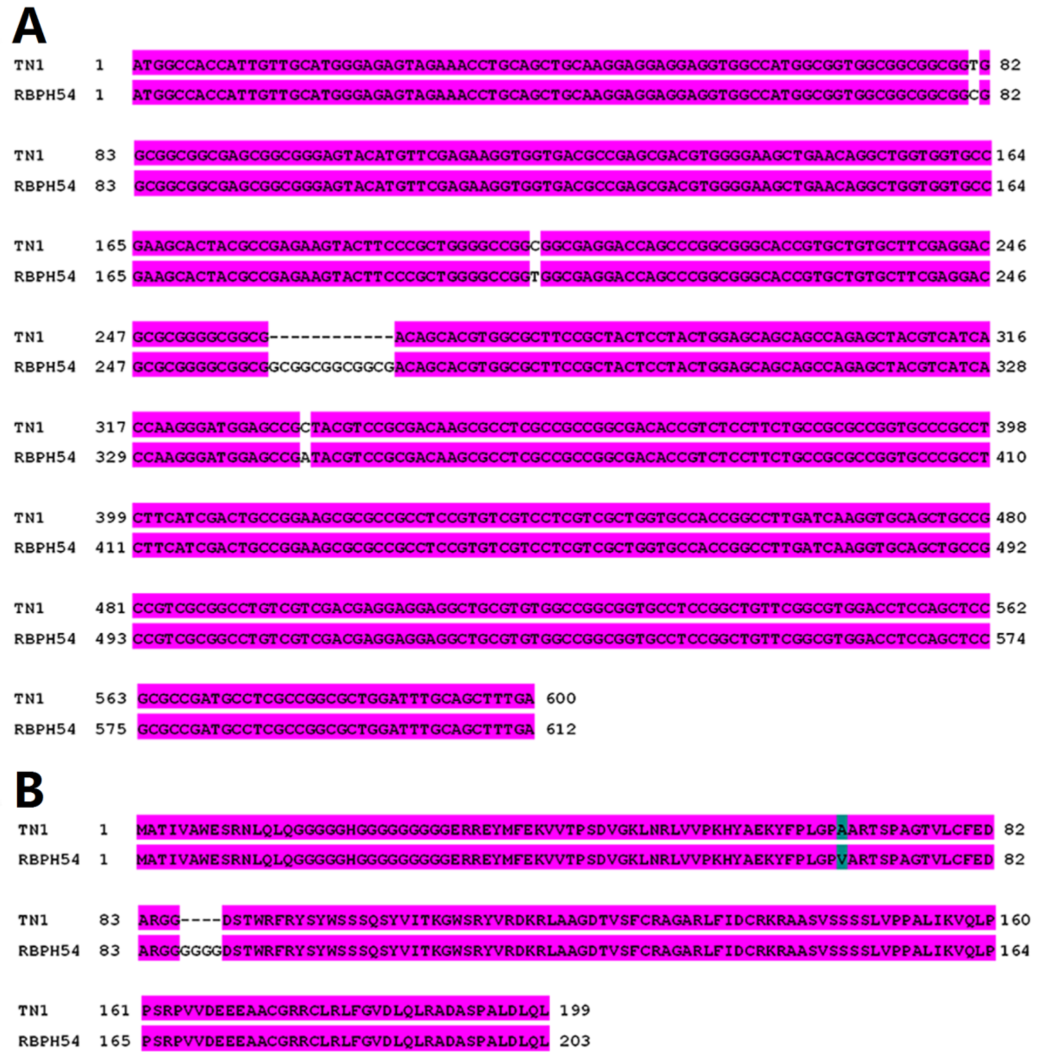

Supplementary Fig. S1. Differences of *G5* between susceptible-parent TN1 and resistant-parent RBPH54.

Alignments of *G5* coding regions cDNA sequences (A) and amino acids sequences (B). The results showed differences included three single nucleotide polymorphism, resulting in one single amino acid mutation from alanine to valine, and a 12-bp insertion corresponding to four glycines in RBPH54. Identical amino acids are in pink, similar amino acids are marked with green.

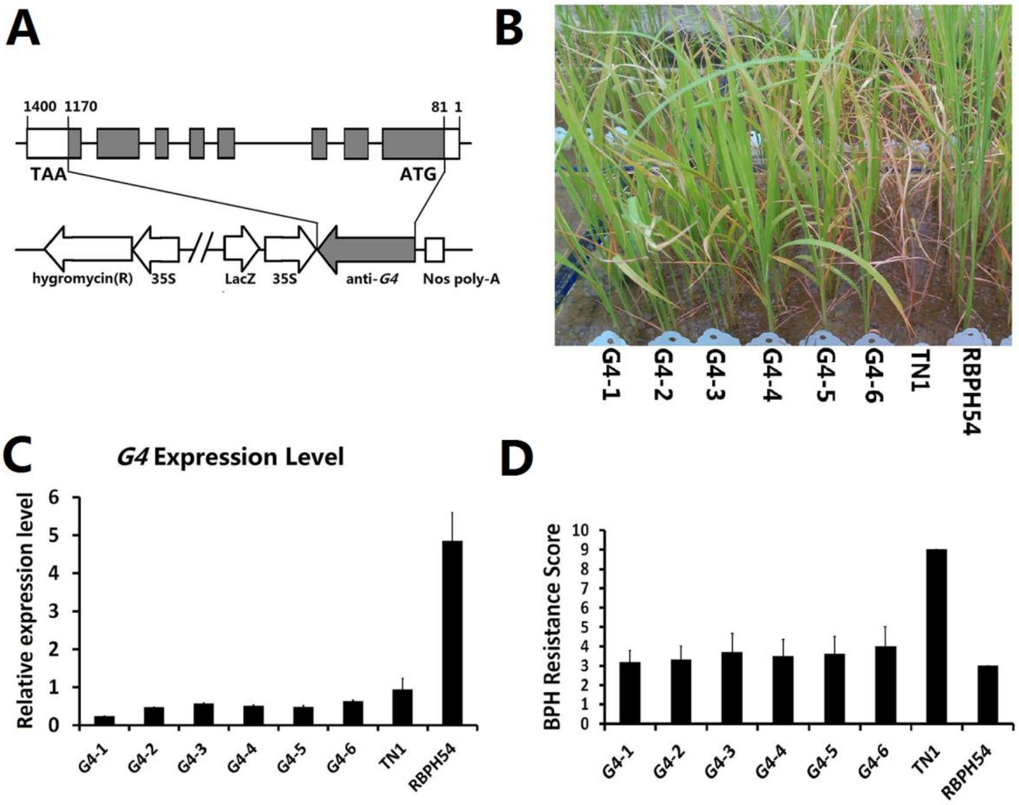

Supplementary Fig. S2. Complementation test for *G4*.

(A) Gene and vector structure of *G4*. The eight exons of *G4* are boxed, with the ORF indicated by a grey box. In the linear map of pCAMBIA1304, *G4* is assembled downstream of the 35S promoter in the antisense form.

(B) BPH-resistance bioassay of *G4*-transgenic lines at the seedling stage. G4-1 to G4-6, 35S::anti-*G4* transgenic T_1 lines; TN1 susceptible-parent control; RBPH54, resistant-parent control.

(C) qRT-PCR analysis of *G4* expression levels in transgenic lines. Data are means ± SD (n = 3 individuals) and expression levels are shown relative to *OsActin1*.

(D) BPH resistance scores of the *G4* transgenic lines. Their lower scores indicate their high resistance to BPH. Data are means ± SD (n = 20 plants). (B) and (D) show that *G4* transgenic lines retained their resistance from RBPH54 after transformation.

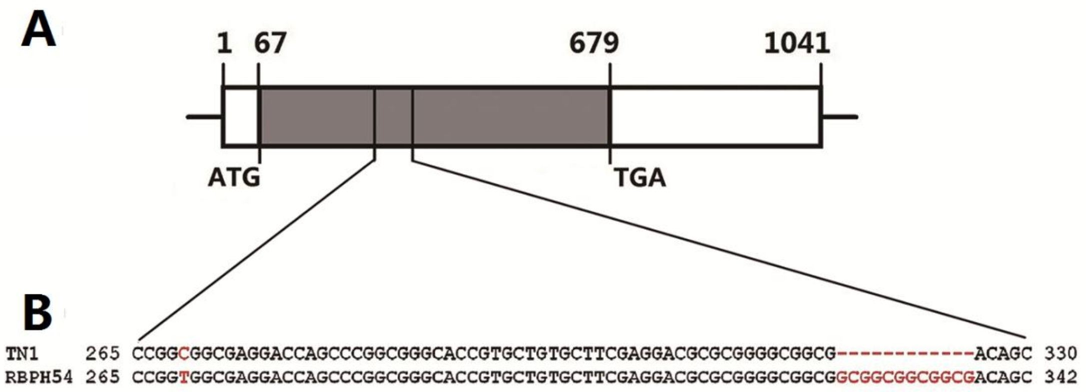

	TN1+/RBPH54+	TN1-/RBPH54+	Independent lines
**G5-a**	8	7	15
**G5-b**	6	6	12

Supplementary Fig. S3. Expression of *G5* alleles in transgenic lines.

(A) Gene structure of *G5*. The ORF is indicated by a grey box, and the region with sequence differences between *G5* dominant and recessive alleles is delineated between the black lines.

(B) The alleles expressed in the *G5* transgenic lines. Both G5-a and G5-b transgenic lines were performed in resistant-parent RBPH54, and the recessive *G5* allele of RBPH54 were consequently detected in all the transgenic lines (RBPH54+). The transgenic lines that dominant *G5* allele of TN1 were also detected (TN1+/RBPH54+) were confirmed as positive transformants and selected to evaluate BPH resistance.

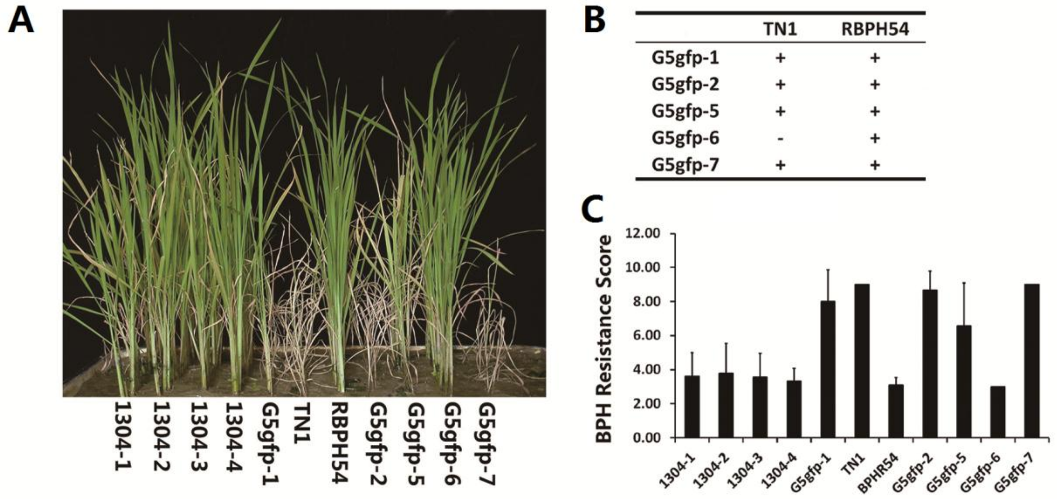

Supplementary Fig. S4. BPH29-GFP fusion protein activity test.

(A) BPH-resistance bioassay of BPH29-GFP fusion protein transgenic lines at the seedling stage. BPH29-GFP fusion protein was assembled into the vector pCAMBIA1304. 1304-1 to 1304-4, 35S::*GFP* transgenic T_1 lines; G5gfp-1 to G5gfp-7, 35S::*BPH29*::*GFP* transgenic T_1 lines; TN1 susceptible-parent control; RBPH54, resistant-parent control.

(B) The alleles expression in the 35S::*BPH29*::*GFP* transgenic lines. As described in Supplementary Fig. S3 legend, the transgenic lines that *G5* allele of both TN1 and RBPH54 were detected (TN1+/RBPH54+) were confirmed as positive transformants. The results corresponded with BPH-resistance bioassay.

(C) BPH resistance scores of BPH29-GFP fusion protein transgenic lines. G5gfp positive transformants lines showed higher scores, indicating their high susceptibility to BPH and the activity of BPH29-GFP fusion protein. The lower scores of 1304 control transgenic lines indicate their high resistance to BPH. Data are means ± SD (*n* = 20 plants).

Supplemental Tables:

Supplemental Table S1. Transcript levels of *BPH29* and *OsActin1* at different rice developmental stages[a]

Number	Sample Name	Signal value[b]			
		BPH29 (Os06g01860)		*OsActin1* (Os03g0718100)	
		Minghui 63	Zhenshan 97	Minghui 63	Zhenshan 97
1	calli, T2, 15 days after induction	9	5	4580	4593
2	calli, T3, 15 days after induction	25	25	6284	4618
3	calli, 15 days after subculture	14	38	4989	3361
4	calli, screening stage	36	29	6657	3912
5	root, seedling with 2 tillers	1574	3242	6759	4570
6	shoot, seedling with 2 tillers	21	24	5915	4254
7	leaf and root at three-leaf stage	102	129	11293	12509
8	flag leaf, 14 days after heading	17	3	2313	2215
9	stem, 5 days before heading	10	19	4992	4614
10	flag leaf, 5 days before heading	26	22	1978	2278
11	stem, heading stage	10	0	7341	4668
12	panicle, heading stage	7	24	10015	6358
13	leaf, 4-5cm young panicle	26	19	4760	2898
14	sheath, 4-5cm young panicle	3	16	3866	3918

15	panicle, 4-5cm young panicle	9	11	5528	4525
16	hull, one day before flowering	16	10	5114	4691
17	stamen, one day before flowering	12	14	21412	8720
18	spikelet, 3 days after pollination	8	10	6496	5176
19	endosperm, 7 days after pollination	19	14	8798	6619

a. Informations obtained from CREP (http://crep.ncpgr.cn).

b. Signal values represent average hybridization signal values. Minghui 63 and Zhenshan 97 are *indica* rice variety.

Supplemental Table S2. Primers used in this work

Purpose	Primer Name	Accession No.	Sequence (5' – 3')	Source
Fine Mapping	RM222		CTTAAATGGGCCACATGCG	Gramene
			CAAAGCTTCCGGCCAAAAG	(http://www.gramene.org/)
	RM244		CCGACTGTTCGTCCTTATCA	Gramene
			CTGCTCTCGGGTGAACGT	(http://www.gramene.org/)
	RM435		ATTACGTGCATGTCTGGCTG	Gramene
			CGTACCTGACCATGCATCTG	(http://www.gramene.org/)
	RM540		GCCTTCTGGCTCATTTATGC	Gramene
			CTAGGCCTGCCAGATTGAAC	(http://www.gramene.org/)
	RM586		ACCTCGCGTTATTAGGTACCC	Gramene
			GAGATACGCCAACGAGATACC	(http://www.gramene.org/)
	RM588		GTTGCTCTGCCTCACTCTTG	Gramene
			AACGAGCCAACGAAGCAG	(http://www.gramene.org/)
	RM589		ATCATGGTCGGTGGCTTAAC	Gramene
			CAGGTTCCAACCAGACACTG	(http://www.gramene.org/)
	BYL7		AAGCTAGGGAATCAGCGGTTA	Yang *et al.*, 2011
			TGTGGCATGTCACTCACTCAC	
	BYL8		CCCACTTCCACAACCACA	Yang *et al.*, 2011

			ATGCTCCTAGCTTCCTATTCC	
	BID2		CACGTAATTATCGCGGTTAC	This study
			TACTAGGCAGGGTTCCTCTC	
	BID3		AAAATTTGAAACACGGGAAT	This study
			GAATATGCTTTGCATCAGTCA	
G4	*G4* qRT-PCR	Os06g01850	ATAATGCTTGCGACCGGTAC	This study
			TGTCAAACTCCTCCTTGTAG	
	G4 Vector		GCACTAGTTTAGTAGACTTCCACGTT	This study
			GCGGTCACCATGGCCGCCGTCACGGCT	
G5	*G5* Sequencing	Os06g01860	ATGGCCACCATTGTTGCATG	This study
			TCAAAGCTGCAAATCCAGCG	
	G5 qRT-PCR		CAAGGGATGGAGCCGATACG	This study
			AAAGCTGCAAATCCAGCGCC	
	G5 Vector		GCACTAGTATGGCCACCATTGTTGCA	This study
			GCGGTCACCTCAAAGCTGCAAATCCAG	
	G5 Promoter		GCGGATCCGCTGGTTCCGAGCTAAGCC	This study
			GCGGTCACCGCTAGATCAAAGCTG	
	Hygromycin test		ATTTGTGTACGCCCGACAGT	This study
			CTCTCGGAGGGCGAAGAATC	

Defense related genes	*OsActin1*	Os03g0718100	TCTGGCATCACACCTTCTACA<br>GGAAGGCTGGAAGAGGAC	This study
	PR10	D38170	CCCTGCCGAATACGCCTAA<br>CTCAAACGCCACGAGAATTTG	Qiu *et al.*, 2007
	PAL	X87946	GCACATCTTGGAGGGAAGCT<br>GCGCGGATAACCTCAATTTG	Du *et al.*, 2009
	CHS	X89859	CCGGCGAACTGCGTGTAC<br>TTCCTGATCTGCGACTTGTCA	Qiu *et al.*, 2007
	NPR1	AY923983	TTTCCGATGGAGGCAAGAG<br>GCTGTCATCCGAGCTAAGTGTT	Du *et al.*, 2009
	AOS2	AY062258	CTCGTCGGAAGGCTGTTGCT<br>ACGATTGACGGCGGAGGTT	Du *et al.*, 2009
	LOX	D14000	GCATCCCCAACAGCACATC<br>AATAAAGATTTGGGAGTGACATATTGG	Qiu *et al.*, 2007
	EIN2	AY396568	CAAGGAACCAGTGACAACCA<br>GCAGTCGTCTCCGCAGTTAG	Du *et al.*, 2009
